# Metabolic disparities in caput, corpus, and cauda epididymis of Hu sheep revealed by LC-MS untargeted metabolomics: implications for sperm maturation

**DOI:** 10.3389/fvets.2025.1680095

**Published:** 2025-12-03

**Authors:** Shujun Shi, Yulan Feng, Lina Zhu, Zhenghan Chen, Qiao Li, Rui Zhang, Xuejiao An, Yaojing Yue

**Affiliations:** 1 Lanzhou Institute of Husbandry and Pharmaceutical Sciences, Chinese Academy of Agricultural Sciences, Lanzhou, China; 2 College of Life Science and Engineering, Northwest Minzu University, Lanzhou, China

**Keywords:** Hu sheep, epididymis, untargeted metabolomics, differential metabolites, sperm maturation

## Abstract

The epididymis is a crucial organ for sperm maturation and storage, with metabolic microenvironmental differences across its functional segments (Caput, Corpus, Cauda) being pivotal to this process. However, the underlying metabolic regulatory mechanisms remain incompletely defined. This study systematically characterized region-specific metabolic profiles in the epididymis of Hu sheep and elucidated their functional associations with sperm maturation. Tissues from the caput, corpus, and cauda of the epididymis from eight Hu rams were subjected to untargeted metabolomic analysis using UHPLC-Q-Exactive HF-X. Differential metabolites were screened by combining multivariate statistical analysis (OPLS-DA, VIP >1) and univariate statistical analysis (student *t*-test, *p* < 0.05), with key metabolic networks identified through KEGG pathway enrichment. We detected 3,393 metabolites, including 1,844 differentially abundant metabolites: 1,038 between the caput and corpus, 1,243 between the cauda and caput, and 1,159 between the cauda and corpus. KEGG analysis revealed significant enrichment in biosynthesis pathways, cAMP signaling, and protein digestion/absorption. Our findings demonstrate that compartmentalized metabolic reprogramming—particularly involving choline and L-carnitine—drives sperm maturation, providing critical targets for optimizing semen cryopreservation technologies in Hu sheep.

## Introduction

The epididymis plays essential roles in sperm motility, transport, maturation, concentration, damage protection, and pre-ejaculatory storage, with distinct functional specialization across its anatomical segments (1). These segments comprise three principal regions: the caput epididymidis (CapE, Caput Epididymidis), corpus epididymidis (CorE, Corpus Epididymidis), and cauda epididymidis (CauE, Cauda Epididymidis) (2, 3). Compartment-specific luminal microenvironments generated by these segments exhibit marked regional heterogeneity, underpinning the functional zonation of the epididymis (4). During transit through these luminal compartments, sperm undergo sequential structural and functional modifications to attain fertilization competence (5). Research indicates that the caput primarily facilitates protein synthesis and secretion, enabling sperm to develop progressive motility essential for ovum penetration (6, 7). The corpus governs sperm maturation through a dynamic secretion-absorption equilibrium maintained by luminal epithelial cells, regulating functional progression until final modifications occur in the distal cauda (8–10). As the primary reservoir for mature sperm, the cauda possesses an expanded lumen where epithelial absorption activity maintains storage stability (11*–*13). Functional specialization across epididymal regions is not only associated with localized gene expression patterns but also dynamically regulated by metabolic microenvironments (13, 14). For example, elevated expression of *SREBP1* and *ELOVL2* in the cauda enhances sperm antioxidant capacity by modulating n-3 polyunsaturated fatty acid (PUFA) synthesis (2). However, systematic characterization of region-specific metabolic profiles and their regulatory mechanisms remains limited. Existing metabolomic studies predominantly focus on the testis or the whole epididymis, with scant attention to segmental metabolic heterogeneity. Moreover, systematic characterization of region-specific metabolic profiles remains notably scarce in high-fecundity livestock breeds such as the Hu sheep. It is plausible that the epididymal microenvironment in such breeds is specifically adapted to sustain their outstanding reproductive performance. As a key Chinese meat breed, the Hu sheep is renowned for its high fecundity, with reported lambing rates of 194.21% in primiparous ewes and an average of 225.21% in multiparous ewes—exceeding 220% based on field data (15). This establishes the Hu sheep as a highly relevant and valuable model for elucidating epididymal metabolic zonation and its contribution to the physiological mechanisms underlying high fertility. Elucidating compartmentalized metabolic signatures in this species addresses two critical gaps: (1) the absence of species-specific metabolic data, and (2) the opportunity to identify novel targets for optimizing *in vitro* sperm preservation technologies. Therefore, a comprehensive analysis of metabolic disparities across epididymal segments in Hu sheep is imperative. This study aims to decode the functional links between regional metabolism and sperm maturation.

Metabolomics has emerged as a crucial tool for elucidating reproductive physiology by comprehensively profiling the dynamic metabolic changes within biological systems. Current metabolomic studies of mammalian reproductive systems predominantly use liquid chromatography-mass spectrometry (LC-MS), gas chromatography–mass spectrometry (GC-MS), and nuclear magnetic resonance (NMR) (16). For instance, a latest study utilizing LC-MS revealed that targeted disruption of the Cabs1gene in mice led to significant alterations in the metabolomic profile of the cauda epididymal lumen fluid, particularly affecting pathways such as arachidonic acid and carnitine metabolism, which ultimately contributed to sperm deformity (17). Similarly, Li et al. (18) demonstrated through untargeted metabolomics that Tibetan sheep adapt to high-altitude hypoxia by modulating metabolites such as adenosine and prostacyclin I2, thereby preserving epididymal function and sperm motility—providing novel insights into the reproductive mechanisms of altitude-adapted species. Despite these advances, a systematic characterization of the region-specific metabolic signatures in the epididymis of high-fertility livestock, such as Hu sheep, remains entirely unexplored.

Building upon these research in epididymal metabolomics (17–19), this study employs an untargeted metabolomics approach, which is particularly suited for this exploratory investigation as it enables a comprehensive, unbiased profiling of the metabolic landscape without pre-defined targets. This approach allows for comparative metabolomic profiling of the caput, corpus, and cauda epididymidis in Hu sheep, integrated with KEGG pathway enrichment analysis, to delineate the spatiotemporal metabolic dynamics underpinning sperm maturation. By synthesizing differential metabolite patterns with functional pathways, we aim to elucidate the compartmentalized metabolic regulatory networks. This approach provides a mechanistic foundation for optimizing sperm preservation techniques in Hu sheep.

## Materials and methods

### Ethics statement

All animal work conducted in the present study was performed in accordance with the guidelines for the care and use of laboratory animals promulgated by the State Council of the People’s Republic of China. This research was approved by the Animal Management and Ethics Committee of Lanzhou Institute of Animal Husbandry and Veterinary Medicine, Chinese Academy of Agricultural Sciences (license number: 2019-008).

### Sample collection and preparation

Testes from eight healthy 6-month-old Hu rams (*Ovis aries*) with comparable body weights were collected at Zhong Sheng Sheep Industry (Huan County, Qingyang, Gansu Province). Samples were transported to the laboratory on ice within 2 h post-collection. Epididymal segments (caput, corpus, cauda) were dissected under sterile conditions in a laminar flow cabinet according to the anatomical criteria defined by Rana et al. (20). To minimize the potential confounding metabolic contributions from luminal spermatozoa and residual fluid, the dissected epididymal segments were gently perfused with ice-cold phosphate-buffered saline (PBS) until the efflux appeared clear. Tissues were sectioned into 2 × 2 cm fragments, transferred to 2 mL cryovials, snap-frozen in liquid nitrogen, and stored at −80 °C prior to metabolomic analysis. In this study, tissue samples from all eight rams were analyzed as individual biological replicates (*n* = 8 independent replicates per epididymal region). All subsequent statistical analyses, including Orthogonal Partial Least Squares-Discriminant Analysis (OPLS-DA) and Kyoto Encyclopedia of Genes and Genomes (KEGG) pathway enrichment analysis, were performed based on this experimental design. The sample size was chosen with reference to published metabolomic studies on similar reproductive tissues (19), ensuring sufficient statistical power to detect significant metabolic differences.

### Reagents and instruments

See Tables 1, 2.

**Table 1:** Standards and reagents.

Name	CAS No.	Purity	Brand
Methanol	67-56-1	Chromatography pure	Merck
Acetonitrile	75-05-8	Chromatography pure	Merck
Acetic acid	64-19-7	Chromatography pure	Rhawn
Ammonium formate	540-69-2	Chromatography pure	Aladdin
Ammonia water	1336-21-6	Chromatography pure	Aladdin
Formic acid	64-18-6	Chromatography pure	Aladdin
Reference standard	—	>98%	Sigma

**Table 2:** Instrument information.

Name	Model	Brand	Place of origin
Mass spectrometer	Q Exactive HF-X	Thermo scientific	Massachusetts, United States
Liquid chromatograph	Vanquish	Thermo scientific	Massachusetts, United States
Centrifuge	5424R	Eppendorf	Hamburg, Germany
Temperature metal mixer	MU-G02-0448	Miulab	Hangzhou, China
Electronic balance	MS105DΜ	Mettler Toledo	Zurich, Switzerland
Centrifugal concentrator	CentriVap	Labconco	Missouri Kansas, United States
Ultrasonic cleaner	KQ5200E	Supmile	Kunshan, China
Pipette	Research plus	Eppendorf	Hamburg, Germany
Automated workstation	Biomek i5	Beckman Coulter	California, United States
Sealing film machine	Mini HES	Monad	Suzhou, China

### Experimental procedures

#### Sample preparation

Tissues from the caput, corpus, and cauda of the epididymis were retrieved from storage at −80 °C and thawed on ice, with all subsequent procedures conducted at 0–4 °C. Tissue homogenization was performed under liquid nitrogen using a cryogenic grinder. Exactly 20 mg of homogenized tissue was weighed into pre-labeled centrifuge tubes, followed by the addition of 400 μL of ice-cold methanol–water extraction solvent (70% methanol, v/v) containing internal standards. The mixtures were vortex-mixed at 1,500 rpm for 5 min and incubated on ice for 15 min. After centrifugation (12,000 × *g*, 10 min, 4 °C), 300 μL of supernatant was transferred to new pre-labeled tubes and stored at −20 °C for 30 min. A second centrifugation step (12,000 × *g*, 3 min, 4 °C) yielded 200 μL of clarified supernatant for LC-MS/MS analysis.

#### Data preprocessing and normalization

Raw mass spectrometry data were converted to mzXML format using ProteoWizard software. Subsequent processing employed XCMS for peak detection, filtration, and alignment. To ensure high data quality, the following thresholds were applied: a mass deviation tolerance of 5 ppm and a signal-to-noise ratio threshold of 3. These values were chosen in accordance with standard practices in untargeted metabolomics: The 5 ppm threshold leverages the high mass accuracy capability of the Q-Exactive HF-X instrument, ensuring confident metabolite identification. The S/N threshold of 3 is a conservative value that robustly distinguishes true metabolite peaks from background noise, minimizing false positives. Background interference was subtracted using blank samples and missing values (occurrence frequency <50%) were imputed via the K-nearest neighbors (KNN) algorithm. To mitigate systematic errors from instrumental drift and sample preparation variations, the raw peak area data underwent a two-step normalization procedure: (1) Internal Standard Calibration using to correct for injection errors and sensitivity fluctuations, followed by (2) Total Ion Current (TIC) Normalization, where each metabolite’s intensity was divided by the sample**’**s total ion current to correct for overall signal variations. The quality of the entire analytical process was monitored by quality control (QC) samples. QC samples were prepared by pooling equal aliquots of all tissue extracts and were injected at intervals of every 10 analytical runs to assess technical reproducibility and system stability. A coefficient of variation (CV) threshold of <0.3 across all QC samples was enforced for any metabolite to be included in the downstream analysis, ensuring data reliability and reproducibility. The resulting preprocessed and normalized data were subjected to downstream metabolite identification and statistical analysis.

#### Metabolite identification and differential metabolite analysis

Metabolite annotation was performed by querying the HMDB, KEGG, and mzCloud public databases. The confidence levels of identification were assigned following a five-tiered system based on the strength of supporting evidence: Level 1a represented the highest confidence, requiring matching with chemical standards for MS1, retention time (RT), and MS/MS spectra; Level 1b indicated a moderate match to standards; Level 2 was assigned for a high-quality match to database records for MS1, RT, and MS/MS; Level 3 denoted a moderate match to database entries; and Level 4, the lowest confidence, was assigned when identification relied solely on MS1 and RT matching—these were excluded from differential analysis. Only metabolites meeting Levels 1 to 3 were retained for subsequent statistical analyses.

Multivariate statistical analyses were conducted using R packages. Principal component analysis (PCA) was first employed to visualize global sample distribution patterns. To statistically validate the clustering patterns observed in PCA, a permutational multivariate analysis of variance (PERMANOVA) was performed. Orthogonal partial least squares-discriminant analysis (OPLS-DA) models were constructed and validated through iterative permutation testing (*n* = 200) to assess model reliability. Differential metabolites were screened by integrating two criteria: (1) variable importance in projection (VIP) scores >1 from the OPLS-DA model, and (2) *p*-values < 0.05 from Student *t*-tests. To control the false discovery rate (FDR) arising from multiple testing, the Benjamini–Hochberg method was applied. Metabolites satisfying both the VIP and *p*-value thresholds were considered candidate differential metabolites. Finally, functionally annotated metabolites were mapped to the KEGG and HMDB databases, and pathway enrichment analysis was conducted using the hypergeometric test.

#### Study scope and limitations

This study employed untargeted metabolomics to profile region-specific metabolic disparities in the epididymis of Hu sheep. It is important to note that no experimental procedures involving semen cryopreservation or functional sperm analysis (e.g., sperm motility assays, membrane integrity tests post-freeze–thaw) were conducted. Therefore, all discussions regarding the potential cryoprotective roles of metabolites such as choline and L-carnitine are derived from metabolomic findings and literature-based inferences, not from direct experimental validation within this study. These hypotheses provide a foundation for future functional studies but should be interpreted as speculative mechanisms awaiting confirmation.

## Results and analysis

### Quality control and total ion chromatogram (TIC) analysis

**Figure 1:**
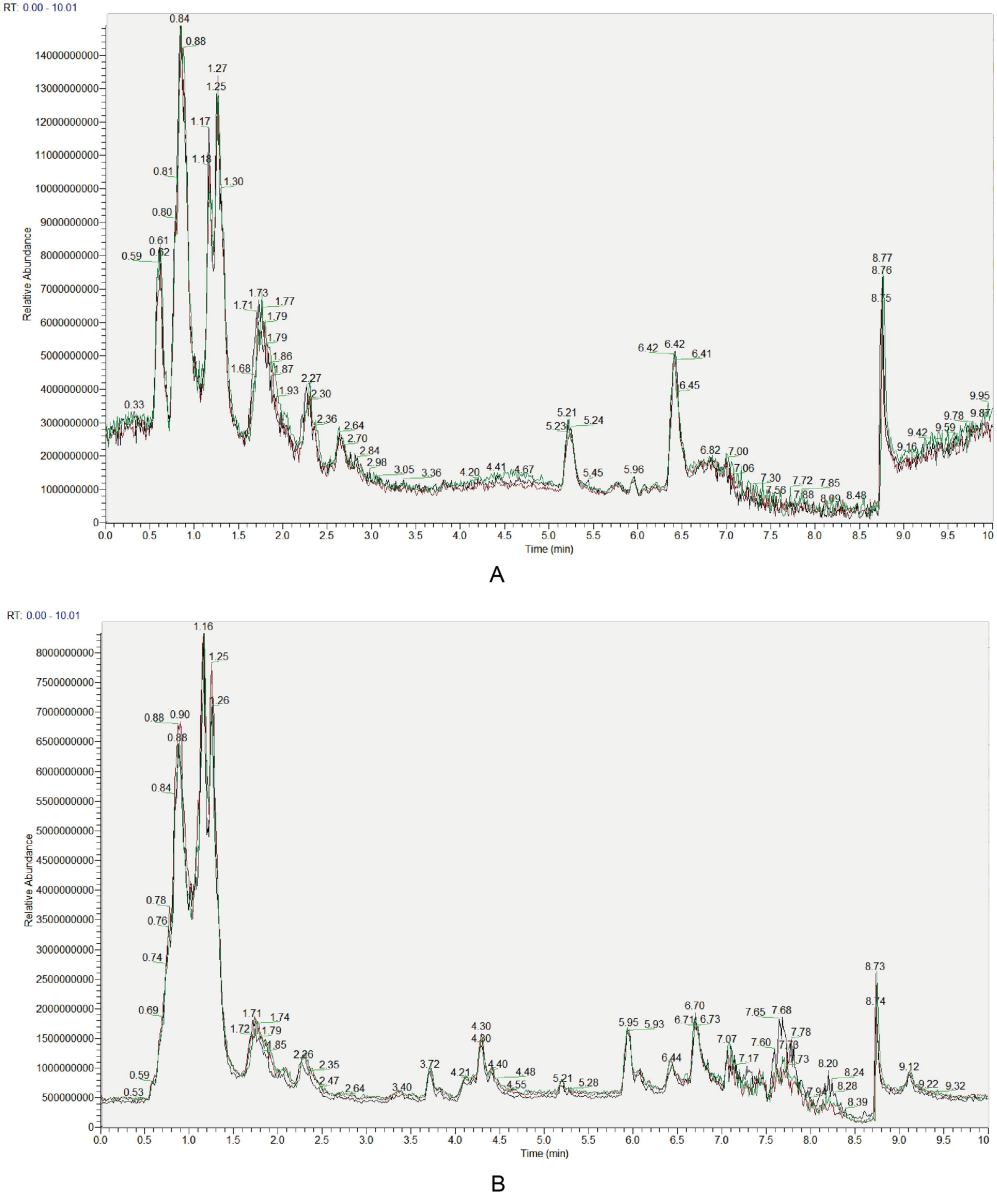
Overlaid TICs of QC samples. **(A)** Positive ionization mode. **(B)** Negative ionization mode.

Quality control (QC) samples were prepared by pooling equal aliquots of tissue extracts from caput, corpus, and cauda epididymidis (*n* = 24). These QC samples were analyzed using identical LC gradients and MS parameters as experimental samples to assess technical reproducibility. Overlaid total ion chromatograms (TICs) from sequential QC injections exhibited high overlap in retention times and peak intensities (Figure 1), indicating excellent signal stability across analytical batches. Meanwhile, to quantitatively evaluate the stability of the liquid chromatography system, we monitored the retention time drift of internal standard compounds in QC samples. Analysis revealed that the coefficient of variation (CV) for all internal standard retention times was below 0.051 (ranging from 0.0415 to 0.0502), indicating that retention time drift was maintained at an extremely low level throughout the sample sequence analysis (far below the commonly accepted 20% threshold). This data robustly demonstrates the stability of the chromatographic conditions, providing crucial assurance for the reliability of metabolite identification.

### Determination results of epididymal metabolites in Hu sheep

**Figure 2:**
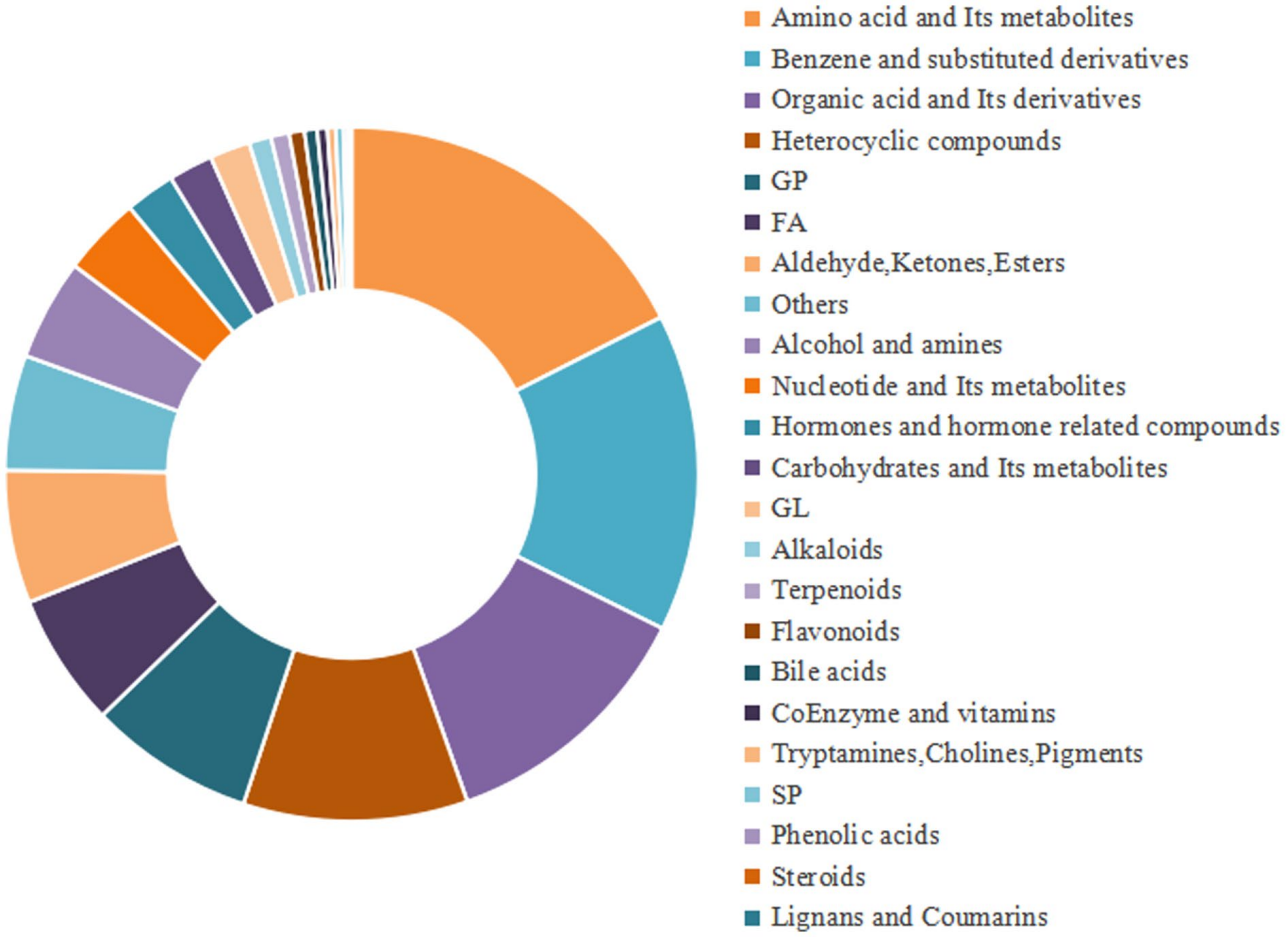
Metabolite classification diagram of all samples.

A total of 3,393 metabolites were detected through non-targeted metabolomics, with 1,721 identified in positive ion mode and 1,672 in negative ion mode. Of these, 92.8% (3,152 out of 3,393) were secondary annotated metabolites. The primary classification revealed that amino acids and their metabolites constituted 17.5%, benzene ring derivatives 14.7%, organic acids and their derivatives 12.2%, heterocyclic compounds 10.4%, and glycerophospholipids 7.7%, making them the main components. In contrast, the low-abundance metabolites included sphingolipids at 0.3%, tryptamine, choline, pigments at 0.3%, phenolic acids at 0.2%, steroids at less than 0.1%, and lignans and coumarins at less than 0.1%, which together accounted for the smallest proportion (refer to Figure 2).

### Principal component analysis of epididymal segments

**Figure 3:**
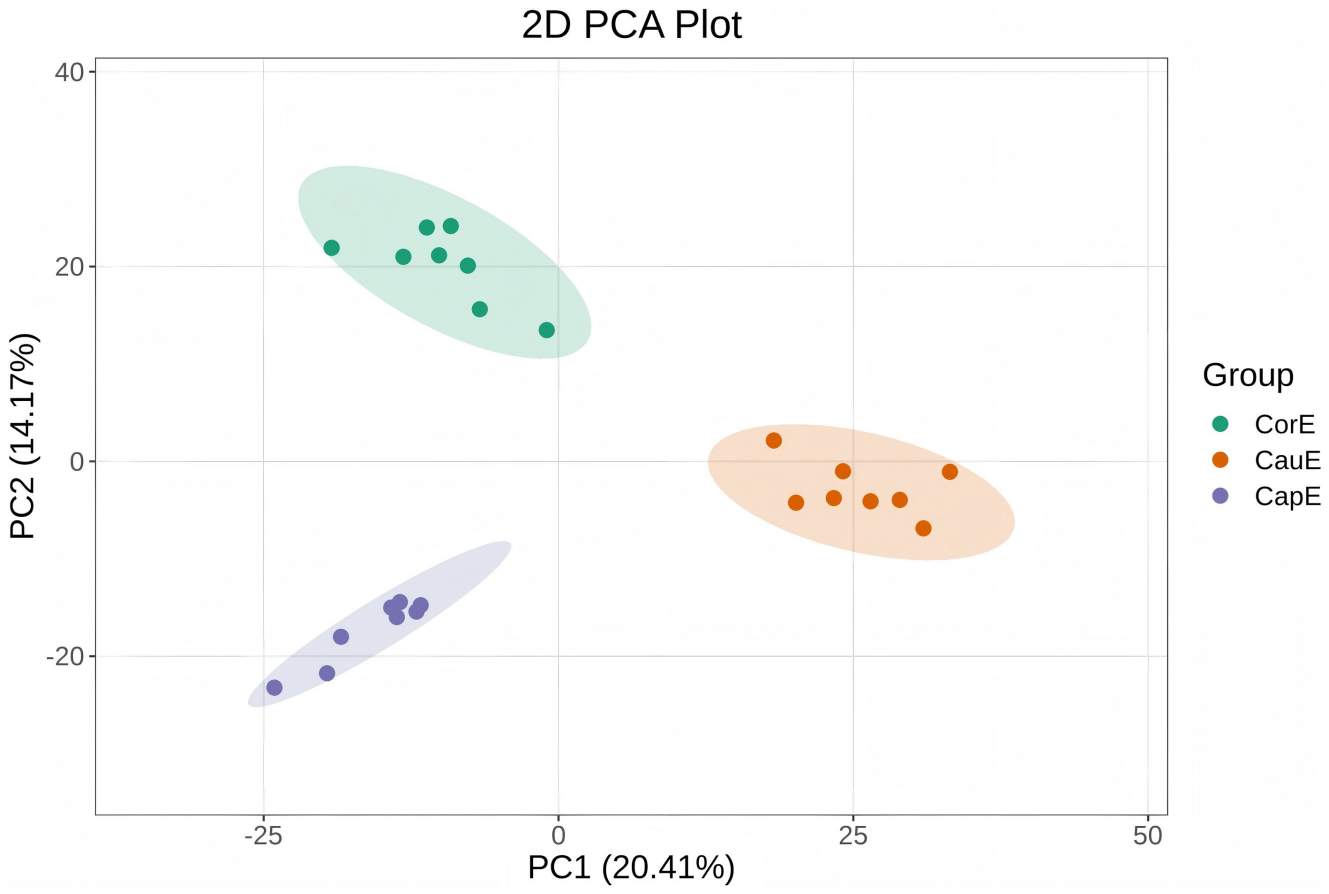
PCA score plot of epididymal segments.

Principal component analysis (PCA) revealed significant spatial segregation of metabolic profiles among the caput (CapE), corpus (CorE), and cauda (CauE) segments. The statistical significance of this clustering pattern was further confirmed by permutational multivariate analysis of variance (PERMANOVA), which demonstrated that the epididymal region was a highly significant factor shaping the metabolic profiles (*R*^2^ = 0.50, *p* = 0.001). The first principal component (PC1) accounted for 20.41% of the total variance, while PC2 explained 14.17%, collectively representing 35.11% of the metabolic heterogeneity. CapE samples predominantly clustered in the left quadrant, whereas CauE samples were distributed in the right quadrant (Figure 3). This distinct compartmentalization confirms a marked metabolic divergence between the caput and cauda regions, underscoring robust experimental reproducibility and analytical reliability.

### Differential metabolite analysis

#### OPLS-DA model validation

OPLS-DA was utilized to analyze the differences in metabolites between the three parts of the Hu sheep epididymis through pairwise comparisons. The goodness of fit (*R*^2^Y) and predictive ability (*Q*^2^) were employed to evaluate the accuracy and predictability of the OPLS-DA model. In this study, the *R*^2^Y and *Q*^2^ values for the comparison between the epididymal head and body were 1 and 0.926, respectively; for the epididymal head versus the epididymal tail, they were 0.999 and 0.947; and for the epididymal tail versus the epididymal body, they were 0.998 and 0.931. All *R*^2^Y and *Q*^2^ values within the model exceeded 0.9, and in 200 random permutation tests, the *Q*^2^ values of the randomized data were significantly lower than the original *Q*^2^ values. This suggests that the established OPLS-DA models possess good accuracy and predictive ability, without any signs of overfitting.

### Differential metabolite profiling

**Figure 4:**
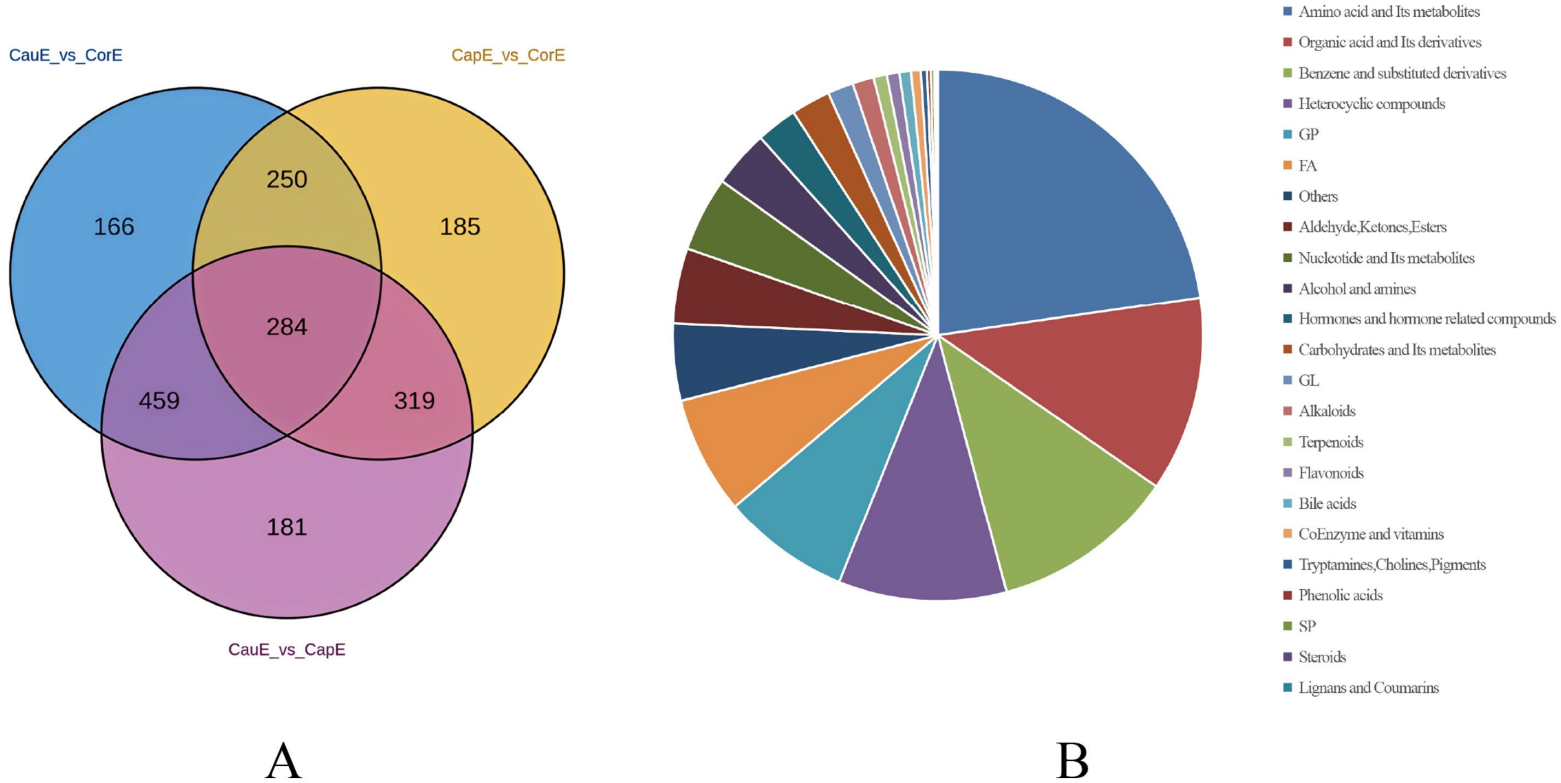
Differential metabolite profiles across epididymal segments in Hu sheep. **(A)** Venn diagram of common metabolites. **(B)** Classification of differential metabolite composition.

**Figure 5:**
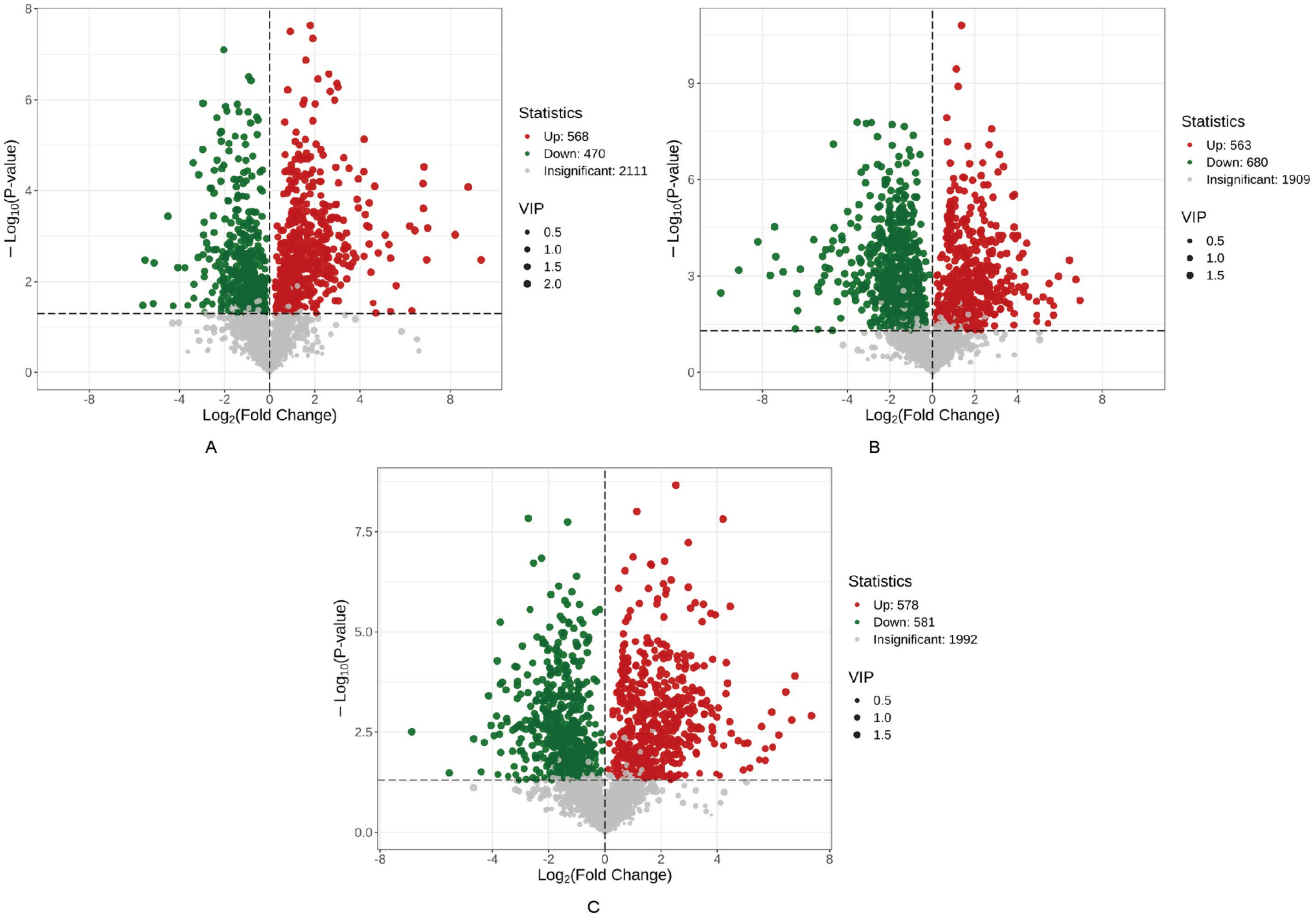
Volcano plot of differential metabolites across epididymal segments in Hu sheep.

Comprehensive differential metabolite analysis identified 1,844 significantly altered metabolites (*VIP* >1, *p* < 0.05) across epididymal segments in Hu sheep, with 284 metabolites common to all three regions (caput, corpus, cauda) (Figure 4A). Amino acids and derivatives constituted the most abundant differential class (419 metabolites), followed by organic acids/derivatives (219), benzene derivatives (207), heterocyclic compounds (189), and glycerophospholipids (144). Conversely, trace differential metabolites included lignans (2), steroids (2), sphingolipids (4), phenolic acids (4), and tryptamine/choline/pigment derivatives (Figure 4B) (7). Pairwise comparisons revealed substantial segment-specific modulation: caput versus corpus exhibited 1,038 differential metabolites (568 upregulated, 470 downregulated in caput) (Figure 5A); cauda versus caput showed 1,243 alterations (563 upregulated, 680 downregulated in cauda) (Figure 5B); while cauda versus corpus displayed 1,159 differentially abundant species (578 upregulated, 581 downregulated in cauda) (Figure 5C). This compartmentalized metabolic reprogramming highlights dynamic functional specialization during sperm maturation.

### KEGG pathway enrichment analysis

**Figure 6:**
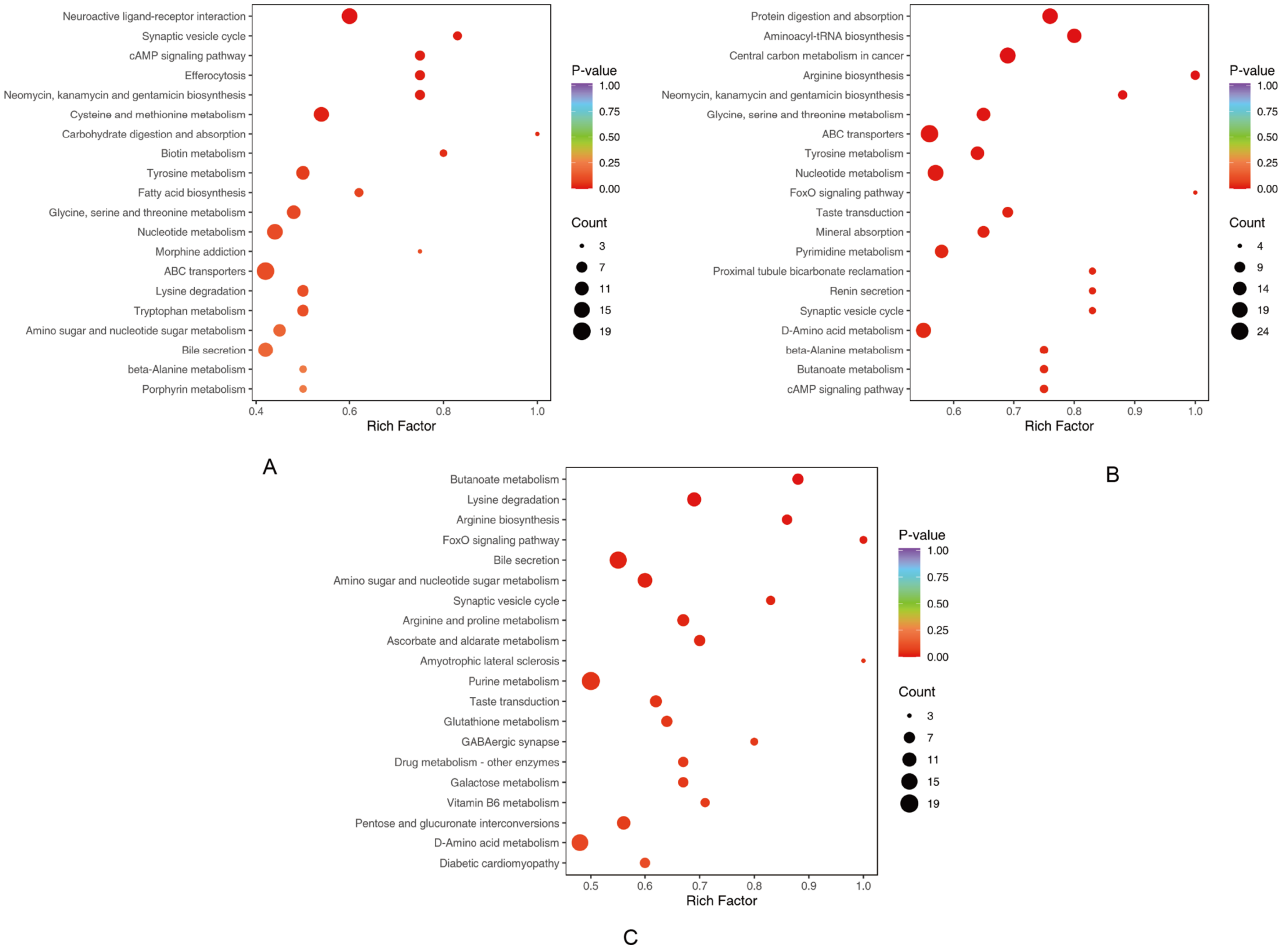
KEGG enrichment of differential metabolites. **(A)** CapE vs. CorE. **(B)** CauE vs. CapE. **(C)** CauE vs. CorE.

To elucidate the biological themes underlying region-specific metabolic variations in the Hu sheep epididymis, we performed KEGG pathway enrichment analysis on the differential metabolites. The top 20 enriched pathways were ranked in ascending order according to their *p*-values. As illustrated in Figure 6, the results revealed that the metabolic reprogramming across the caput, corpus, and cauda regions was predominantly orchestrated around three core biological themes: amino acid and nucleotide turnover, central carbon and energy metabolism, and endocrine and signaling regulation. The comparative analysis between the caput and corpus regions highlighted a significant emphasis on neuroactive ligand-receptor interactions and cAMP-mediated signaling, pointing to active signal transduction mechanisms (Figure 6A). Conversely, comparisons involving the cauda region, such as cauda vs. caput and cauda vs. corpus, were strongly characterized by pathways related to protein digestion and absorption, aminoacyl-tRNA biosynthesis, and arginine biosynthesis, underscoring this region’s role in substrate processing and preparation for sperm maturation (Figures 6B,C). Additionally, pathways like butanoate metabolism and FoxO signaling were notably enriched, further implicating energy metabolism and cellular stress response in the functional specialization of the epididymis. This thematic summary demonstrates a compartmentalized yet interconnected metabolic network supporting the distinct physiological functions of each epididymal segment.

## Discussion

As a Chinese indigenous breed with high fecundity, Hu sheep exhibit widely reported low post-thaw sperm motility, significantly limiting artificial insemination applications (21). Although cryopreservation enables long-term storage of superior sperm to enhance breeding efficiency, its effectiveness remains suboptimal in ovine species. The epididymis—a pivotal site for sperm maturation—harbors compartmentalized metabolic microenvironments whose dynamic regulatory mechanisms are incompletely understood, as highlighted in broader metabolomic studies of the epididymis (5, 22). Our comparative metabolomics approach identifies region-specific metabolites with membrane-stabilizing and antioxidant functions, thereby providing mechanistic insights for optimizing cryopreservation protocols in Hu sheep. Our study addresses this gap by employing a comparative metabolomics approach to identify region-specific metabolites with membrane-stabilizing and antioxidant functions. These findings not only map the metabolic landscape but also provide mechanistic insights into how the epididymal microenvironment supports sperm maturation, thereby offering a rational foundation for optimizing cryopreservation protocols in Hu sheep.

Employing LC-MS-based untargeted metabolomics, we characterized 3,393 metabolites across caput, corpus, and cauda epididymidis in Hu sheep. Dominant metabolic classes included amino acids/derivatives, benzene derivatives, organic acids/derivatives, heterocyclic compounds, and glycerophospholipids. Multivariate analysis (PCA and OPLS-DA) identified 1,844 differentially abundant metabolites exhibiting region-specific distribution gradients, with amino acids, organic acids, and glycerophospholipids showing the most pronounced compartmentalization. These spatially defined metabolic gradients are not merely observational; they strongly suggest that a highly coordinated program of energy metabolism reprogramming and membrane remodeling constitutes the central driving force behind post-testicular sperm maturation (17, 23). This active, epithelium-driven programming of the luminal milieu ensures that spermatozoa are sequentially exposed to the specific metabolites required for their functional development at each epididymal segment, a phenomenon that appears to be an evolutionarily conserved strategy for ensuring sperm competence, as evidenced in other mammalian models (24*–*26).

KEGG enrichment analysis revealed significant functional divergence in metabolic networks across epididymal segments. Our metabolomic data showed a pronounced enrichment of choline (VIP = 1.8; level 1a confidence) in the caput epididymidis. As a water-soluble vitamin B complex component, choline deficiency impairs mitochondrial function and induces oxidative stress (27, 28). As a crucial precursor for phosphatidylcholine synthesis, choline is fundamental for regulating sperm membrane fluidity and maturation (29, 30). The accumulation of choline in the caput region, as identified in our study, suggests the establishment of a metabolic reservoir that supports post-testicular sperm maturation. This finding is consistent with the intriguing hypothesis proposed by Hanley et al. (27), which suggests that genital tract-derived choline could be utilized by sperm for autocrine/paracrine cholinergic signaling to drive motility. The involvement of the neuroactive ligand-receptor interaction pathway in our KEGG results further supports the potential role of cholinergic mechanisms in fertilization (31). Moreover, the cryoprotective properties of choline, documented during phosphatidylcholine synthesis (32, 33), alongside its strategic enrichment in the epididymis revealed in our study, highlight its potential as an endogenous cryoprotectant. The physiological positioning of this choline reservoir provides a rationale for exploring its application in optimizing sperm preservation techniques for Hu semen.

Concurrently, arginine demonstrated significant enrichment in the caput (4.48-fold higher than the cauda; *VIP* = 1.46; level 1a confidence), which contributes to sperm activation (34, 35). This pronounced gradient suggests a targeted provision of arginine by the caput epithelium, creating a reservoir for post-ejaculatory sperm function. Li et al. (34) expanded on this, showing that food-derived arginine-rich peptides stimulated spermatogenesis recovery in busulfan-treated mice. Sahoo et al. (35) documented that L-arginine treatment markedly boosted sperm motility and triggered capacitation in goat spermatozoa. Critically, these *in vivo* and *in vitro* findings are robustly supported by interventional studies. Administration of L-arginine was proven to significantly ameliorate busulfan-induced oligospermia in rats, effectively restoring sperm count, motility, and velocity by mitigating oxidative stress (MDA reduction), suppressing apoptosis (caspase-3 downregulation), and restoring cellular energy levels (ATP elevation) (36). Mechanistically, nitric oxide derived from arginine (via nitric oxide synthase, NOS) counters hydrogen peroxide (H₂O₂)-induced apoptosis by suppressing lipid peroxidation and upregulating anti-apoptotic Bcl-2 expression (37, 38). This action safeguards membrane integrity and cellular function (39). Practical applications across species support this: arginine-supplemented cryodiluents enhance post-thaw semen quality in pigs and donkeys (40*–*42). The combined evidence—from its native epididymal abundance and multifaceted mechanisms to its proven efficacy in mitigating reproductive toxicity and improving cryosurvival—strongly warrants its evaluation as a cryoprotectant candidate for Hu sheep.

The corpus epididymidis of Hu sheep precisely regulates sperm maturation through a dynamic secretion-absorption equilibrium in luminal epithelial cells, involving metabolic reprogramming (e.g., glycolysis-dominated energy supply) and signaling pathway activation (e.g., cAMP-PKA axis) (43, 44). This process occurs within a specialized immunomodulatory microenvironment, as evidenced by studies in ruminants where the epididymal epithelium actively manages immune responses to prevent autoimmunity while defending against pathogens (45). Notably, localized inflammatory microenvironments and potential pathogen invasion during sperm maturation may compromise functional integrity (46, 47). Therefore, maintaining a delicate immunometabolic balance is critical for preserving male fertility, especially since reproductive tract infections are known to severely compromise sperm quality in rams (48). Our metabolomics revealed significant enrichment of metabolites annotated to the neomycin/kanamycin/gentamicin biosynthesis pathway (KEGG enrichment) in the corpus. A key ion feature contributing to this enrichment (VIP = 1.65; 3.89-fold vs. caput) was putatively annotated as kanamycin (level 3 confidence). This suggests the activation of an intrinsic antibiotic biosynthesis pathway in the corpus epididymidis. We hypothesize that the production of kanamycin-like structural analogs or related antimicrobial compounds may contribute to microenvironmental homeostasis through dual potential mechanisms: sperm membrane stabilization via phospholipid binding and broad-spectrum antimicrobial protection against Gram-negative bacteria, which aligns with findings that epididymal immune cells (e.g., macrophages and dendritic cells) in ruminants exhibit region-specific antimicrobial functions (49). The discovery of this antibiotic biosynthetic potential in the Hu sheep epididymis highlights a potential species-specific adaptation for maintaining sterility in a high-sperm-production breed, possibly compensating for the heightened inflammatory risks associated with prolonged sperm storage. Collectively, these actions may preserve sperm viability and ensure functional maturation, underscoring the evolutionary refinement of reproductive tract immunity in Hu sheep.

As the principal sperm reservoir, the cauda epididymidis maintains fertility during extended sperm storage through profound lipid metabolic remodeling and robust antioxidant defenses (50, 51). Our metabolomics revealed a remarkable accumulation of acetyl-L-carnitine (*VIP* = 1.57; 2.62-fold compared to the caput; level 1a confidence) and L-carnitine (*VIP* = 1.67; 16.28-fold compared to the caput; level 1a confidence) within this region. This extreme gradient—likely represents a key physiological adaptation supporting the prolonged sperm storage capability essential for the high fecundity of Hu sheep. These findings align with established transporter-mediated accumulation mechanisms, wherein epithelial cells in the caput and corpus actively uptake and secrete these compounds into the lumen, culminating in a peak concentration within the cauda (52, 53). At the molecular level, this process is facilitated by specific carnitine transporters, which are highly expressed in the epididymal epithelium and are crucial for maintaining the luminal carnitine concentration essential for sperm function (54).

Functionally, this carnitine reservoir is poised to support sperm on multiple fronts. Within sperm mitochondria, it promotes fatty acid *β*-oxidation, supplying energy for maturation and post-ejaculatory motility activation (55). Beyond its role as an energy substrate, L-carnitine exhibits multifaceted protective properties relevant to the cauda’s storage environment. It can scavenge reactive oxygen species (ROS) to suppress lipid peroxidation and DNA fragmentation, and contributes to stabilizing membrane structures (56, 57). The central role of carnitine is further underscored by studies showing that defects in its transport systems directly impair sperm energy production (58). Therefore, the exceptional abundance of carnitine we identified in the cauda epididymidis highlights its potential as a crucial component of the endogenous microenvironment that safeguards sperm during storage. This discovery provides a strong physiological rationale for exploring its application. The empirical evidence that exogenous L-carnitine supplementation improves post-thaw semen quality in other species collectively positions it as a highly promising candidate for optimizing semen preservation protocols in Hu sheep (59, 60).

## Conclusion

In conclusion, our study identifies compartmentalized metabolic reprogramming along the Hu sheep epididymis, with 1,844 region-specific differential metabolites (*VIP* > 1, *p* < 0.05) forming pronounced gradients in amino acids, organic acids, and glycerophospholipids. Importantly, we pinpoint choline and L-carnitine as key metabolites with region-specific enrichment patterns that provide a mechanistic foundation for potentially improving sperm cryosurvival. While these findings suggest innovative avenues for improving semen cryopreservation protocols, any cryoprotective applications remain speculative until validated through functional experiments. This work primarily establishes a metabolic foundation for future research in reproductive biotechnology.

## Data Availability

The data presented in the study are deposited in the OMIX, China National Center for Bioinformation repository, accession number OMIX013206.

## Supplementary material

Supplementary Figure S1 OPLS-DA validation plot. The permutation test plot (200 permutations) evaluates the robustness of the Orthogonal Projections to Latent Structures Discriminant Analysis (OPLS-DA) model.

## References

[1] Breton S Nair AV Battistone MA. Epithelial dynamics in the epididymis: role in the maturation, protection, and storage of spermatozoa. Andrology. (2019) 7:631–43. doi: 10.1111/andr.12632, 31044554 PMC6688936

[2] Liu JM. Study on fatty acid composition and antioxidant properties of epididymal cauda in Hu sheep with different fertility [dissertation]. Lanzhou, China: Lanzhou University (2022).

[3] Belleannée C Calvo E Thimon V Cyr DG Légaré C Garneau L . Role of microRNAs in controlling gene expression in different segments of the human epididymis. PLoS One. (2012) 7:e34996. doi: 10.1371/journal.pone.0034996, 22511979 PMC3325285

[4] Zhu WQ Zhang XM. Research progress on epididymal structure and function. Chin J Vet Sci. (2021) 41:1195–209. doi: 10.16303/j.cnki.1005-4545.2021.06.24

[5] Cornwall GA. New insights into epididymal biology and function. Hum Reprod Update. (2009) 15:213–27. doi: 10.1093/humupd/dmn055, 19136456 PMC2639084

[6] Nixon B De Iuliis GN Hart HM Zhou W Mathe A Bernstein IR . Proteomic profiling of mouse epididymosomes reveals their contributions to post-testicular sperm maturation. Mol Cell Proteomics. (2019) 18:S91–S108. doi: 10.1074/mcp.RA118.000946, 30213844 PMC6427233

[7] Zhang HN Zhao ZW Wei JH Li Z. Research advances on epididymosomes-mediated sperm maturation. Chin J Reprod Contracept. (2022) 42:974–9. doi: 10.3760/cma.j.cn101441-20210813-00346

[8] Gervasi MG Visconti PE. Molecular changes and signaling events occurring in spermatozoa during epididymal maturation. Andrology. (2017) 5:204–18. doi: 10.1111/andr.12320, 28297559 PMC5354101

[9] Battistone MA Spallanzani RG Mendelsohn AC Capen D Nair AV Brown D . Novel role of proton-secreting epithelial cells in sperm maturation and mucosal immunity. J Cell Sci. (2020) 133:jcs233239. doi: 10.1242/jcs.233239, 31636115 PMC7003979

[10] Johnston DS Jelinsky SA Bang HJ DiCandeloro P Wilson E Kopf GS . The mouse epididymal transcriptome: transcriptional profiling of segmental gene expression in the epididymis. Biol Reprod. (2005) 73:404–13. doi: 10.1095/biolreprod.105.039719, 15878890

[11] Turner TT. Resorption versus secretion in the rat epididymis. J Reprod Fertil. (1984) 72:509–14. doi: 10.1530/jrf.0.0720509, 6512773

[12] Elfgen V Mietens A Mewe M Hau T Middendorff R. Contractility of the epididymal duct: function, regulation and potential drug effects. Reproduction. (2018) 156:R125–41. doi: 10.1530/REP-17-0754, 30304934

[13] O’Flaherty C. Orchestrating the antioxidant defenses in the epididymis. Andrology. (2019) 7:662–8. doi: 10.1111/andr.12630 31044545

[14] Michael A. Functional specificity of genes in the caput, corpus and cauda epididymidis of yak-cattle hybrids based on RNA sequencing] [Dissertation]. Sichun, China: Southwest University of Science and Technology (2021).

[15] Pan Y. Study on the large-scale breeding model, growth and development, and reproductive performance of Hu sheep [dissertation]. Xianyang, China: Northwest A&F University (2018).

[16] Gan L Hu Y Lian Y Wang XZ. Research progress of metabolomics in function of mammal testis. Chinese Journal of Veterinary Science, (2019) 39:1410–5. doi: 10.16303/j.cnki.1005-4545.2019.07.30

[17] Zhao X Nie J Zhou W Zeng X Sun X. The metabolomics changes in epididymal lumen fluid of CABS1 deficient male mice potentially contribute to sperm deformity. Front Endocrinol. (2024) 15:1432612. doi: 10.3389/fendo.2024.1432612, 39234505 PMC11371703

[18] Li Y Yang Y Yu B Gao R Wang X. Transcriptome and metabolome analyses reveal high-altitude adaptation mechanism of epididymis sperm maturation in Tibetan sheep. Animals. (2024) 14:3117. doi: 10.3390/ani14213117, 39518841 PMC11544902

[19] Yao RY. Screening of differential metabolites and mechanism study affecting testicular development in Hu sheep [dissertation. Lanzhou, China: Lanzhou University (2022).

[20] Rana M Roy SC Divyashree BC. Sperm antioxidant defences decrease during epididymal transit from caput to cauda in parallel with increases in epididymal fluid in the goat. Reprod Fertil Dev. (2017) 29:1708–19. doi: 10.1071/RD16269 27677348

[21] Wang YY Zhang H Chen SK Wang BY Dong HS. Study on the difference of sperm quality and antioxidant properties between black-boned sheep and Hu sheep after semen cryopreservation and resuscitation. Chinese Journal of Animal Science, (2025) 61:249-52+61. doi: 10.19556/j.0258-7033.20240614-04

[22] Lv C Wu G Hong Q Quan G. Spermatozoa cryopreservation: state of art and future in small ruminants. Biopreserv Biobank. (2019) 17:171–82. doi: 10.1089/bio.2018.0113, 30499684

[23] Collodel G Castellini C Lee JC Signorini C. Relevance of fatty acids to sperm maturation and quality. Oxidative Med Cell Longev. (2020) 2020:7038124. doi: 10.1155/2020/7038124, 32089776 PMC7025069

[24] Zhou W De Iuliis GN Dun MD Nixon B. Characteristics of the epididymal luminal environment responsible for sperm maturation and storage. Front Endocrinol. (2018) 9:59. doi: 10.3389/fendo.2018.00059, 29541061 PMC5835514

[25] Ren X Jiang K Yin J Ma Z Chen Z Yang K . Integrated transcriptomic and metabolomic analysis of goose epididymis reveals molecular markers associated with sperm mobility. Poult Sci. (2025) 104:105180. doi: 10.1016/j.psj.2025.105180, 40273680 PMC12051571

[26] Yao R Zhao P Ma H Li W Weng X Li F . Analyses of widely targeted metabolic profiling reveal enhanced energy metabolism in well-developed testicular tissue of Hu sheep. Domest Anim Endocrinol. (2025) 91:106909. doi: 10.1016/j.domaniend.2024.106909, 39729915

[27] Hanley PJ. Elusive physiological role of prostatic acid phosphatase (PAP): generation of choline for sperm motility via auto-and paracrine cholinergic signaling. Front Physiol. (2023) 14:1327769. doi: 10.3389/fphys.2023.1327769, 38187135 PMC10766772

[28] Glade MJ Crook MA. Choline deficiency: is it being recognized? Nutrition. (2022) 94:111509. doi: 10.1016/j.nut.2021.111509, 34862116

[29] Zhang Y Liang C Zhang J Wang N Zheng H Wang J. Choline supplementation alleviates fluoride-induced testicular toxicity by restoring the NGF and MEK expression in mice. Toxicol Appl Pharmacol. (2016) 310:205–14. doi: 10.1016/j.taap.2016.09.018, 27664006

[30] Karakus C Ozyurt R. Correlation between high choline metabolite signal in spectroscopy and sperm retrieval chance at micro-TESE. Eur Rev Med Pharmacol Sci. (2022) 26:1125–30. doi: 10.26355/eurrev_202202_28102, 35253167

[31] Zhan X Fletcher L Dingle S Baracuhy E Wang B Huber LA . Choline supplementation influences ovarian follicular development. Front Biosci (Landmark Ed). (2021) 26:1525–36. doi: 10.52586/5046, 34994167

[32] Xu B Bai X Zhang J Li B Zhang Y Su R . Metabolomic analysis of seminal plasma to identify goat semen freezability markers. Front Vet Sci. (2023) 10:1132373. doi: 10.3389/fvets.2023.1132373, 36968471 PMC10036599

[33] Sicchieri F Silva AB Santana VP Vasconcelos MAC Ferriani RA Vireque AA . Phosphatidylcholine and L-acetyl-carnitine-based freezing medium can replace egg yolk and preserves human sperm function. Transl Androl Urol. (2021) 10:397–407. doi: 10.21037/tau-20-1004, 33532327 PMC7844480

[34] Liu W Zhang L Gao A Khawar MB Gao F Li W. Food-derived high arginine peptides promote spermatogenesis recovery in busulfan-treated mice. Front Cell Dev Biol. (2021) 9:791471. doi: 10.3389/fcell.2021.791471, 34993200 PMC8724571

[35] Sahoo B Gupta MK. Effect of arginine-induced motility and capacitation on RNA population in goat spermatozoa. Vet Res Commun. (2023) 47:1427–44. doi: 10.1007/s11259-023-10092-3, 37162640

[36] Abd-Elrazek AM Ahmed-Farid OAH. Protective effect of L-carnitine and L-arginine against busulfan-induced oligospermia in adult rat. Andrologia. (2018) 50:e12806. doi: 10.1111/and.12806, 28444774

[37] Suschek CV Schnorr O Hemmrich K Aust O Klotz LO Sies H . Critical role of L-arginine in endothelial cell survival during oxidative stress. Circulation. (2003) 107:2607–14. doi: 10.1161/01.CIR.0000066909.13953.F1, 12742995

[38] Liang MC Yang L. Antioxidant mechanism of arginine. Chinese Journal of Bioinformatics. (2020) 18:201–5. doi: 10.12113/202007001

[39] Hou GC. Effects of L-arginine on liquid storage of boar semen and its mechanism [dissertation. Xianyang, China: Northwest A&F University (2021).

[40] Huang ZY Yang QX Yang HW Yang R Wu Y. Effects of arginine on boar semen quality at different storage temperatures. Modern Animal Husbandry Science and Technology. (2025) 7:59–62. doi: 10.19369/j.cnki.2095-9737.2025.07.016

[41] Wu G Bazer FW Davis TA Jaeger LA Johnson GA Kim SW . Important roles for the arginine family of amino acids in swine nutrition and production. Livest Sci. (2007) 112:8–22. doi: 10.1016/j.livsci.2007.07.003

[42] Zhao J Li JD. Effects of arginine and bovine serum albumin in diluents on frozen donkey semen quality. Heilongjiang Anim Sci Vet Med. (2022) 18:68–72+141. doi: 10.13881/j.cnki.hljxmsy.2021.12.0093

[43] Barrachina F Battistone MA Castillo J Mallofré C Jodar M Breton S . Sperm acquire epididymis-derived proteins through epididymosomes. Hum Reprod. (2022) 37:651–68. doi: 10.1093/humrep/deac015, 35137089 PMC8971652

[44] Yuan M. Metabolic reprogramming during spermatogenesis [dissertation]. Xiamen, China: Xiamen University (2022).

[45] Rodriguez AR Babcock RL Guimarães JPT Kaur G Dufour JM. Immune regulation in the testis and epididymis. Adv Exp med biol. (2025) 1469:25–47. doi: 10.1007/978-3-031-82990-1_2 40301251

[46] Oghbaei H Rezaei YR Nikanfar S Zarezadeh R Sadegi M Latifi Z . Effects of bacteria on male fertility: spermatogenesis and sperm function. Life Sci. (2020) 256:117891. doi: 10.1016/j.lfs.2020.117891 32504760

[47] Hasan H Bhushan S Fijak M Meinhardt A. Mechanism of inflammatory associated impairment of sperm function, spermatogenesis and steroidogenesis. Front Endocrinol. (2022) 13:897029. doi: 10.3389/fendo.2022.897029, 35574022 PMC9096214

[48] Zhang M-F Wan S-C Chen W-B Yang D-H Liu W-Q Li B-L . Transcription factor Dmrt1 triggers the SPRY1-NF-κB pathway to maintain testicular immune homeostasis and male fertility. Zool Res. (2023) 44:505–21. doi: 10.24272/j.issn.2095-8137.2022.440, 37070575 PMC10236308

[49] Zhao H Yu C He C Mei C Liao A Huang D. The immune characteristics of the epididymis and the immune pathway of the epididymitis caused by different pathogens. Front Immunol. (2020) 11:2115. doi: 10.3389/fimmu.2020.02115, 33117332 PMC7561410

[50] James ER Carrell DT Aston KI Jenkins TG Yeste M Salas-Huetos A. The role of the epididymis and the contribution of epididymosomes to mammalian reproduction. Int J Mol Sci. (2020) 21:5377. doi: 10.3390/ijms21155377, 32751076 PMC7432785

[51] Devi A Kushwaha B Maikhuri JP Singh R Gupta G. Cell signaling in sperm midpiece ensures quiescence and survival in cauda epididymis. Reproduction. (2021) 162:339–51. doi: 10.1530/REP-21-0202, 34486982

[52] Agarwal A Sengupta P Durairajanayagam D. Role of L-carnitine in female infertility. Reprod Biol Endocrinol. (2018) 16:5. doi: 10.1186/s12958-018-0323-4, 29373970 PMC5785901

[53] Longo N Frigeni M Pasquali M. Carnitine transport and fatty acid oxidation. Biochim Biophys Acta. (2016) 1863:2422–35. doi: 10.1016/j.bbamcr.2016.01.023, 26828774 PMC4967041

[54] Li D Liu J Du W Liu H Xiao W Song X . Carnitine/organic cation transporter 2 (OCTN2) contributes to rat epididymal epithelial cell growth and proliferation. Biomed Pharmacother. (2017) 93:444–50. doi: 10.1016/j.biopha.2017.06.057, 28666211

[55] Naderi Noreini S Malmir M Ghafarizadeh A Faraji T Bayat R . Protective effect of L-carnitine on apoptosis, DNA fragmentation, membrane integrity and lipid peroxidation of spermatozoa in asthenoteratospermic men. Andrologia. (2021) 53:e13932. doi: 10.1111/and.13932 33368462

[56] Koohpeyma F Siri M Allahyari A Mahmoodi M Saki F Dastghaib S. The effects of L-carnitine on renal function and gene expression of caspase-9 and Bcl-2 in monosodium glutamate-induced rats. BMC Nephrol. (2021) 22:162. doi: 10.1186/s12882-021-02364-4 33933022 PMC8088661

[57] Heidari M Qasemi-Panahi B Moghaddam G Daghigh-Kia H Masoudi R. L-carnitine improves quality parameters and epigenetic patterns of buck's frozen-thawed semen. Anim Reprod Sci. (2022) 247:107092. doi: 10.1016/j.anireprosci.2022.107092, 36306715

[58] Kuang W Zhang J Lan Z Deepak RNVK Liu C Ma Z . SLC22A14 is a mitochondrial riboflavin transporter required for sperm oxidative phosphorylation and male fertility. Cell Rep. (2021) 35:109025. doi: 10.1016/j.celrep.2021.109025, 33882315 PMC8065176

[59] Tatemoto H Osokoshi N Hirai M Masuda Y Konno T Yamanaka K. Addition of l-carnitine to the freezing extender improves post-thaw sperm quality of Okinawan native Agu pig. Theriogenology. (2022) 188:170–6. doi: 10.1016/j.theriogenology.2021.12.030, 35031142

[60] Ramón-López AE Fernández-Collahuazo JP Samaniego JX Duma JM Méndez MS Soria ME . L-carnitine supplementation in conventional slow and ultra-rapid freezing media improves motility, membrane integrity, and fertilizing ability of dog epididymal sperm. Anim Reprod Sci. (2024) 270:107580. doi: 10.1016/j.anireprosci.2024.10758 39216207

