# Supplementary figures and images for: Metabolic disparities in caput, corpus, and cauda epididymis of Hu sheep revealed by LC-MS untargeted metabolomics: implications for sperm maturation
Source: Front Vet Sci. 2025 Dec 3;12:1680095. doi: 10.3389/fvets.2025.1680095 (PMC12709674; doi:10.3389/fvets.2025.1680095)

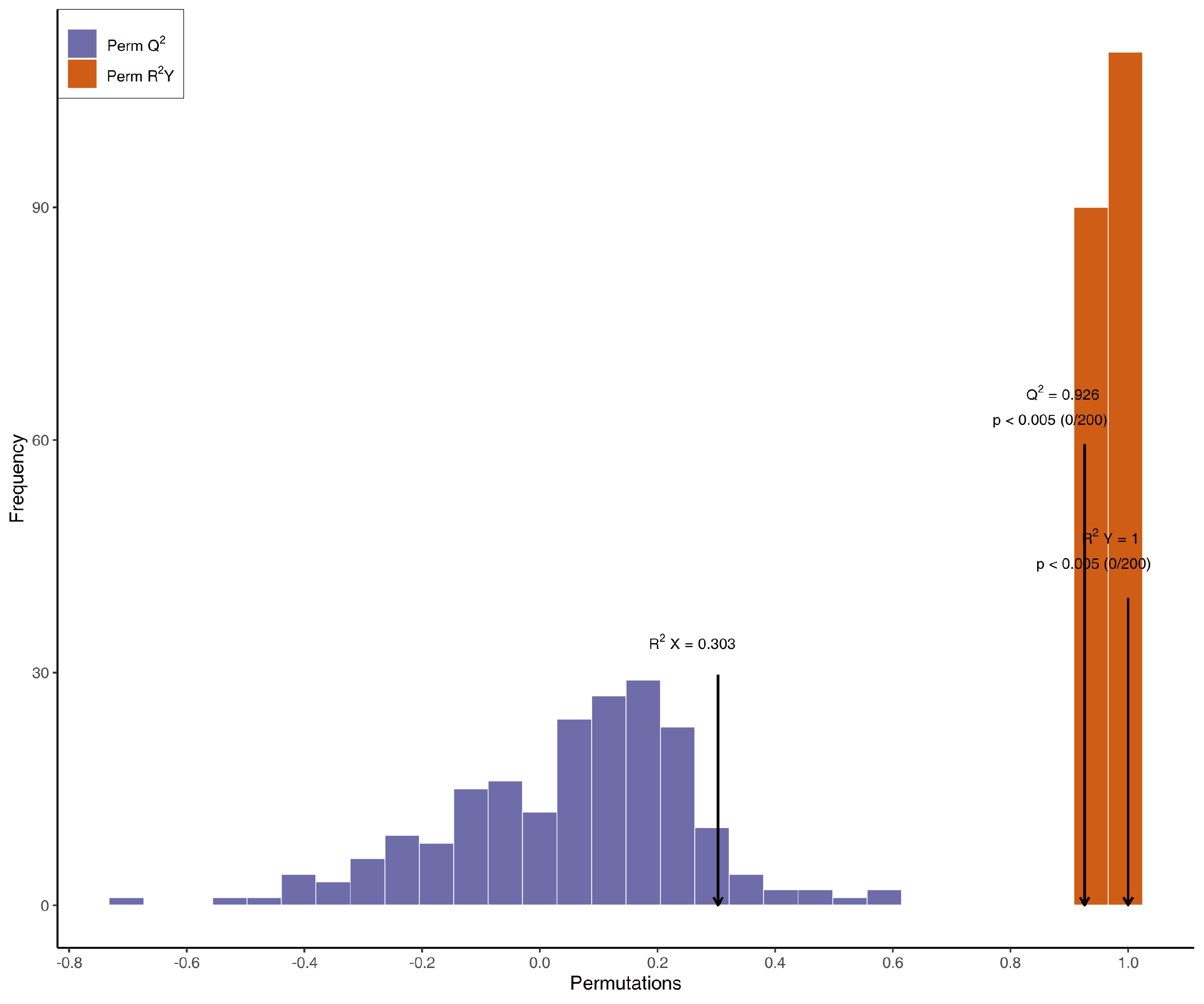

Supplement: Supplementary Figure S1 — OPLS-DA validation plot. The permutation test plot (200 permutations) evaluates the robustness of the Orthogonal Projections to Latent Structures Discriminant Analysis (OPLS-DA) model. [file Image_1.tif]
